# Advanced lung cancer inflammation index predicts survival outcomes of hepatocellular carcinoma patients receiving immunotherapy

**DOI:** 10.3389/fonc.2023.997314

**Published:** 2023-03-14

**Authors:** Qian Li, Fei Ma, Ju feng Wang

**Affiliations:** ^1^ Department of Oncology, The Affiliated Cancer Hospital of Zhengzhou University & Henan Cancer Hospital, Zhengzhou, China; ^2^ Department of General Surgery, The Affiliated Cancer Hospital of Zhengzhou University & Henan Cancer Hospital, Zhengzhou, China

**Keywords:** advanced lung cancer inflammatory index (ALI), hepatocellular carcinoma, Immunotherapy, nomogram, prognosis

## Abstract

**Objective:**

We evaluate the predictive significance of the Advanced Lung Cancer Inflammation Index (ALI) in patients with advanced hepatocellular carcinoma (HCC) following therapy with immune checkpoint drugs.

**Methods:**

In 2018-2020, 98 patients with advanced hepatocellular carcinoma who were treated with immune checkpoint inhibitors at our hospital were compiled. Using the receiver operating characteristic (ROC) curve, the appropriate cut-off point for ALI was determined. Kaplan-Meier analysis, the Cox proportional hazards model, and Nomogram plots highlighted the relationship between ALI and overall survival (OS). The model was validated using calibration plots, receiver operating characteristic curves (ROC), and decision curve analysis (DCA), which was performed on 52 patient sets by external validation.

**Results:**

The AUC for ALI was 0.663. The best cutoff value was 36.5, with a median overall survival (OS) of 473 days for patients with ALI≤ 36.5 and 611 days for those with ALI > 36.5. Univariate analysis revealed that the presence or absence of local treatment, alpha-fetoprotein (AFP), and ALI were prognostic factors; LASSO regression analysis identified four candidate variables. Multifactorial COX analysis revealed that high ALI was an independent prognostic factor for overall survival in both groups (HR = 0.411; 95% CI: 0.244-0.651; P<0.001). In addition, the Nomogram model that included ALI was able to predict the success of immunotherapy in patients with advanced liver cancer more accurately.

**Conclusion:**

ALI is a novel prognostic marker in immunotherapy-treated patients with advanced hepatocellular cancer.

## Background

Hepatocellular carcinoma (HCC) is the fifth most prevalent malignancy worldwide, with the third highest fatality rate. Hepatocellular carcinoma accounts for 85-90 percent of primary liver cancer (1, 2). Sixty to seventy percent of patients with early-stage HCC experience recurrence or distant metastases within five years, despite the fact that surgery continues to be the primary therapy option. In addition, early indications of liver cancer are atypical, and when the disease is discovered, it is frequently intermediate or advanced, with 90% of patients unable to undergo surgery. Therefore, it is crucial to investigate more effective therapy options for advanced HCC (3).

Systemic chemotherapy is the conventional treatment for hepatocellular carcinoma, but the effect of chemotherapy for hepatocellular carcinoma in clinical practice is unsatisfactory due to the low sensitivity of hepatocellular carcinoma to chemotherapy and the liver injury of hepatocellular carcinoma patients, which affects the metabolism of chemotherapy drugs (4). Immunotherapy for hepatocellular carcinoma has risen to the forefront of research in recent years, and the main immunotherapy medications are immune checkpoint inhibitors, primarily programmed cell death protein-1 (PD-1) and programmed cell death ligand-1 (PD-L1). Immunotherapy alone and immunotherapy in combination with targeted medicines are increasingly employed to treat advanced liver cancer (5). However, there are no established, practical, and reliable prognostic markers for immunotherapy patients.

Cancer growth, tumorigenesis, and metastasis are caused by systemic inflammation and malnutrition, and there is mounting evidence that inflammation plays a vital role in the progression of cancer (6). It has been established that the presence of a systemic inflammatory response is related to the clinical prognosis of a number of cancers (7). Therefore, inflammation-based biomarkers such as C-reactive protein albumin ratio (CAR), platelet-to-lymphocyte ratio (PLR), neutrophil-to-lymphocyte ratio (NLR), and albumin-globulin ratio (AGR) have been used for early assessment of prognosis in a variety of tumours (8, 9). In 2013, Jafri et al. combined NLR, serum albumin levels, and body mass index (BMI) into a subsequently, it was discovered that ALI may be beneficial for predicting survival outcomes in different tumours (10, 11). However, the prognostic utility of this biomarker in immunotherapy for patients with advanced hepatocellular carcinoma remains unclear. We examined the association between ALI and the clinical features and prognosis of patients with advanced hepatocellular carcinoma by analysing retrospectively clinical data from patients treated with immune checkpoint drugs.

## Methods

### Study design

This retrospective study involved 98 patients with advanced hepatocellular carcinoma who were treated with PD-1 inhibitors between February 2018 and February 2019. On the patients, case data collection and telephone follow-up were undertaken. For validation, an additional 52 liver cancer patients were gathered between April 2019 and April 2020 using the identical entrance row criteria.

Patients who met the diagnostic criteria for HCC, were at the BCLC-C stage, were between 18 and 80 years old, and had complete clinical and follow-up data were enrolled. Exclusion criteria: Patients with concomitant significant disorders of other systems, such as myocardial infarction, cerebral infarction, renal failure, or patients with metastatic liver cancer or multiple tumours, post-transplantation, pregnancy, or breastfeeding.

Diagnostic criteria: In this investigation, the diagnostic criteria for HCC were based on the regulations of the American Association for the Study of Liver Diseases (AASLD). For staging liver cancer, the BCLC 2010 staging criteria were utilised, and patients with advanced HCC were classified as BCLC-C.

### Laboratory procedures

Patients’ height, weight, and blood samples were collected within the first two weeks of treatment. ALI = BMI x Alb/NLR, where BMI = weight (kg)/(height (m)^2^), Alb = serum albumin (g/dL), and NLR = absolute neutrophil count/absolute lymphocyte count.

### Statistical analysis

The connection between clinicopathological factors and ALI was analysed using the χ2 test and Wilcoxon test. Using the Kaplan-Meier method, survival curves were created and compared using the log-rank test. Overall survival (OS) was measured from the date the patient received his or her first immunotherapy treatment until the date of death from any cause or the date of the final contact with the patient. Disease-free survival (DFS) was defined as the interval between the date of the patient’s first immunotherapy and the date of disease recurrence, all-cause death, or the date on which the patient was last contacted. The training set consisted of 98 patients with advanced liver cancer who were analysed using the R caret software. For external validation, an additional 52 individuals with liver cancer from other hospitals were recruited. With the glmnet programme, LASSO (last absolute shrinkage and selection operator) regression analysis was conducted. In order to acquire a subset of predictors, LASSO regression analysis minimises the prediction error of the quantitative response variables by constraining the model parameters so that the regression coefficients of selected variables approach zero. The LASSO regression analysis was conducted in R using 10-fold cross-validation, and the optimal lambda values were then chosen. lambda.lse produced a model with good performance and the fewest number of independent variables. The variables identified by the LASSO regression model were then incorporated into the prediction model by multi-factor logistic regression analysis.

The preceding analysis was performed using SPSS (version 20.0) and R software (version 4.0.2). A statistically significant difference was determined to exist when p<0.05.

### Treatment and patient follow up

After hospital release, all patients were evaluated for cancer recurrence and metastasis using tumour marker measures every three months and abdominal ultrasonography, CT, or magnetic resonance imaging every six months. The time limit for patient follow-up was set at 36 months after therapy.

## Results

### Baseline characteristics

This retrospective analysis included 98 patients with clinical stage BCLC-C, including 66 men and 32 women with a mean age of 52 years and a median survival time of 462 days. 54 patients had HBV, 2 patients had HCV, 42 patients had non-viral hepatitis, and 74 patients died. 39.8% of patients received topical treatment, while 33 (33.7%) and 65 (66.3%) were treated with immunosuppressive medications in first and second line, respectively. The patient population shown above served as the training set. 52 patients with advanced liver cancer were re-collected at a different time periods using the aforementioned validation set criteria. All patients completed the necessary examinations, and **Table 1** outlines the fundamental characteristics of the two groups.

**Table 1:** Characteristics of clinical data in the modeling and validation groups.

Variable	Development cohort	Validation cohort	p
N	98	52	
Gender, n (%)			0.037
F	32 (21.3%)	8 (5.3%)	
M	66 (44%)	44 (29.3%)	
Viral Hepatitis, n (%)			1.000
HBV	54 (36%)	29 (19.3%)	
HCV	2 (1.3%)	1 (0.7%)	
None	42 (28%)	22 (14.7%)	
ECOG, n (%)			0.943
0	38 (25.3%)	19 (12.7%)	
1	48 (32%)	27 (18%)	
2	12 (8%)	6 (4%)	
Child-Pugh class, n (%)			0.950
A	81 (54%)	42 (28%)	
B	17 (11.3%)	10 (6.7%)	
Local treatment, n (%)			0.564
0	59 (39.3%)	28 (18.7%)	
1	39 (26%)	24 (16%)	
Immunotherapy, n (%)			0.686
First-line	33 (22%)	20 (13.3%)	
Second-line	65 (43.3%)	32 (21.3%)	
Age, meidan (IQR)	52 (48, 59)	52 (47.75, 59.5)	0.803
ALT, meidan (IQR)	37 (27.25, 58)	30 (20.5, 57.5)	0.036
AST, meidan (IQR)	47 (27, 80.75)	44 (24.75, 80.75)	0.665
OS, meidan (IQR)	462 (400.5, 586.75)	375 (300, 465.5)	< 0.001
AFP, median (IQR)	60 (30, 86.75)	63.5 (28.75, 88)	0.895
ALI, median (IQR)	36.54 (23.34, 49.42)	34.36 (17.74, 47.74)	0.098

### ROC diagrams for ALI and its constituents

**Figure 1:**
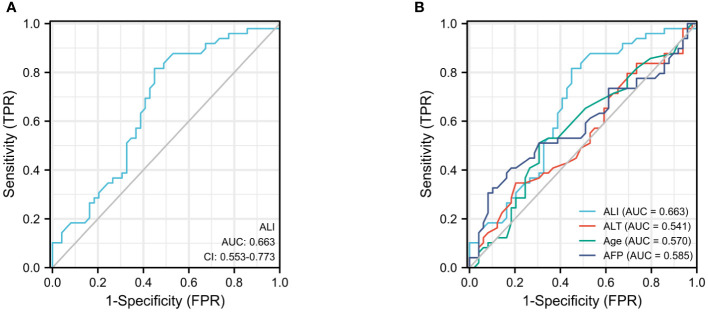
The ROC curves for ALI **(A)**, AFP, ALT, and age **(B)** in advanced hepatocellular carcinoma patients.

Using the continuous variable ALI as the test variable and median survival 462 days (15.2 months) as the state variable, the subject operating characteristic (ROC) curve was calculated to be 0.663 (95%CI:0.553-0.775, P=0.005), and the cutoff value for ALI was determined to be 36.5 (sensitivity: 72.7%; specificity: 53.8%) (**Figure 1A**). Patients were separated into high ALI (n = 48) and low ALI (n = 50) groups. In the meantime, we computed the ROC curves of additional clinical indicators such as AFP, ALT, and AST on OS and discovered that their predictive values were all inferior than those of ALI (**Figure 1B**).

### Analysis of survival according to ALI subset

**Figure 2:**
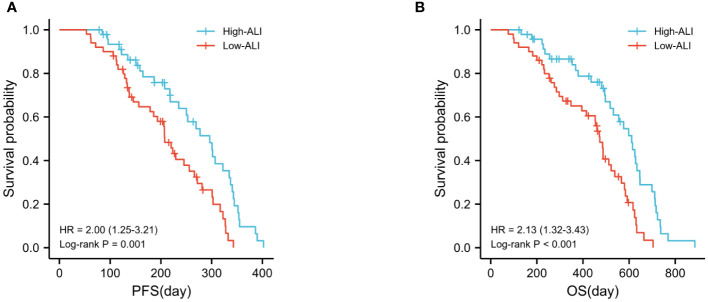
Kaplan–Meier analysis of PFS **(A)** and OS **(B)** in relation to the ALI in HCC.

The high ALI group had a considerably greater overall survival rate than the low ALI group (p<0.0001). The median progression-free survival (PFS) and overall survival (OS) were 207 and 473 days, respectively, for patients with ALI 36.5, compared to 296 and 611 days for patients with ALI ≤36.5 (**Figures 2A, B**).

### Relationship between several subgroups of ALI and its constituents and clinicopathological variables

The association between ALI and its components and clinicopathological variables is displayed in **Table 2**. In terms of baseline clinical data features, there was no significant difference between groups with high and low ALI. Nonetheless, it was linked to overall patient survival. The group with a high ALI had a longer total survival time than the group with a low ALI.

**Table 2:** The correlations between the ALI and the clinicopathological factors.

Variable	ALI (≤36.5)	ALI (>36.5)	p
N	50	48	
Gender, n (%)			0.722
F	15 (15.3%)	17 (17.3%)	
M	35 (35.7%)	31 (31.6%)	
Viral Hepatitis, n (%)			0.201
HBV	30 (30.6%)	24 (24.5%)	
HCV	2 (2%)	0 (0%)	
None	18 (18.4%)	24 (24.5%)	
ECOG, n (%)			0.502
0	19 (19.4%)	19 (19.4%)	
1	23 (23.5%)	25 (25.5%)	
2	8 (8.2%)	4 (4.1%)	
Child-Pugh class, n (%)			0.131
A	38 (38.8%)	43 (43.9%)	
B	12 (12.2%)	5 (5.1%)	
Local treatment, n (%)			0.322
0	33 (33.7%)	26 (26.5%)	
1	17 (17.3%)	22 (22.4%)	
Immunotherapy, n (%)			0.777
First-line	18 (18.4%)	15 (15.3%)	
Second-line	32 (32.7%)	33 (33.7%)	
Age, meidan (IQR)	52 (48, 60.5)	52 (48.75, 58.25)	0.912
ALT, meidan (IQR)	35.5 (26.25, 56)	39 (29, 59.5)	0.332
AST, meidan (IQR)	45 (27.75, 77.5)	48 (25, 86.75)	0.529
OS, meidan (IQR)	441 (378.5, 545.75)	493 (416.25, 627.25)	0.004
AFP, meidan (IQR)	46.5 (29.25, 79.5)	65 (31.5, 103)	0.346
ALI, meidan (IQR)	23.45 (18.54, 33.34)	49.55 (40.26, 55.05)	< 0.001

### Analysis of univariate and multifactor COX regressions

Age, gender, Child-Pugh score, history of hepatitis, alanine aminotransferase (ALT) level and ECGO score, and number of immunotherapy lines had no statistically significant effect on OS prognosis. Patients with a high ALI score had a significantly prolonged OS (HR, 0.411; 95% CI, 0.244-0.693; P< 0.001, **Table 3**).

**Table 3:** Univariate and Multivariate analysis of poor prognostic factors for OS in HCC patients.

Clinical characteristics	N	Univariate		Multivariate	
		HR (95% CI)	P value	HR (95% CI)	P value
Gender	98				
F	32	Reference			
M	66	0.700 (0.427-1.147)	0.157		
Viral Hepatitis	98				
None	42	Reference			
HBV	54	0.892 (0.548-1.452)	0.645		
HCV	2	1.067 (0.251-4.528)	0.930		
ECOG	98				
0	38	Reference			
1	48	1.051 (0.651-1.696)	0.839		
2	12	0.684 (0.238-1.968)	0.481		
Child-Pugh Class	98				
A	81	Reference			
B	17	1.368 (0.742-2.523)	0.316		
Local treatment	98				
None	59	Reference			
Done	39	0.457 (0.280-0.744)	**0.002**	0.366 (0.212-0.630)	**<0.001**
Immunotherapy	98				
First-line	33	Reference			
Second-line	65	0.912 (0.545-1.527)	0.727		
ALI	98				
≤36.5	50	Reference			
>36.5	48	0.406 (0.243-0.678)	**<0.001**	0.411 (0.244-0.693)	**<0.001**
Age	98				
<50	28	Reference			
≥50	70	0.832 (0.496-1.394)	0.485		
AFP	98				
<20	18	Reference			
≥20	80	2.072 (1.127-3.807)	**0.019**	2.582 (1.359-4.904)	**0.004**
ALT	96				
<40	54	Reference			
≥40	42	0.906 (0.564-1.453)	0.681		

### Predictive model development

**Figure 3:**
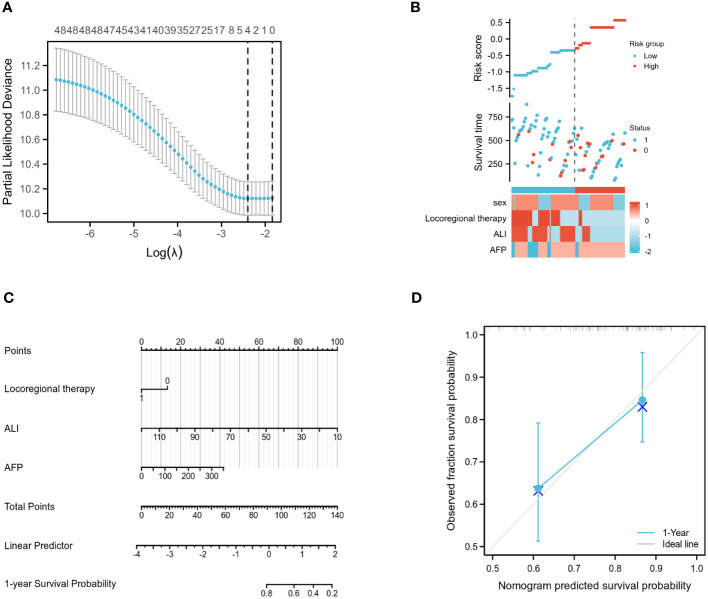
Training set model construction. **(A)** LASSO binary logistic regression models were used for variable selection. **(B)** Models screened for risk factor visualization. **(C)** Nomogram for predicting OS in patients with advanced hepatocellular carcinoma based on ALI and independent prognostic factors. **(D)** Nomogram calibration chart for 1-year OS prediction in advanced liver cancer patients.

In this work, LASSO regression analysis was utilised to exclude predictor variables from **Table 1** prior to developing a prediction model using multifactor logistic regression. Four of the original variables, namely gender, AFP level, whether or not topical therapy was administered, and ALI group score, were incorporated in the prediction model as predictors (**Figure 3A**). The LASSO regression model contained coefficients for these four variables that were not zero (**Figure 3B**).

Multifactor COX analysis revealed that the independent prognostic factors affecting OS were AFP level, whether or not local therapy had been administered, and ALI group score. Using the R programming language, the aforementioned variables were merged to generate a Nomogram prediction model for patients with advanced liver cancer after immunotherapy (**Figure 3C**). The associated scores were determined by projecting the points of each variable onto the “Points” axis, and the corresponding scores were added to get the total score corresponding to the projected outcome. The receiver operating characteristic (ROC) curve analysis revealed that the Concordance (C-index) of this Nomogram model was 0.720 (95% CI: 0.686-0.753, p<0.01) (**Figure 3D**).

### Predictive model validation

**Figure 4:**
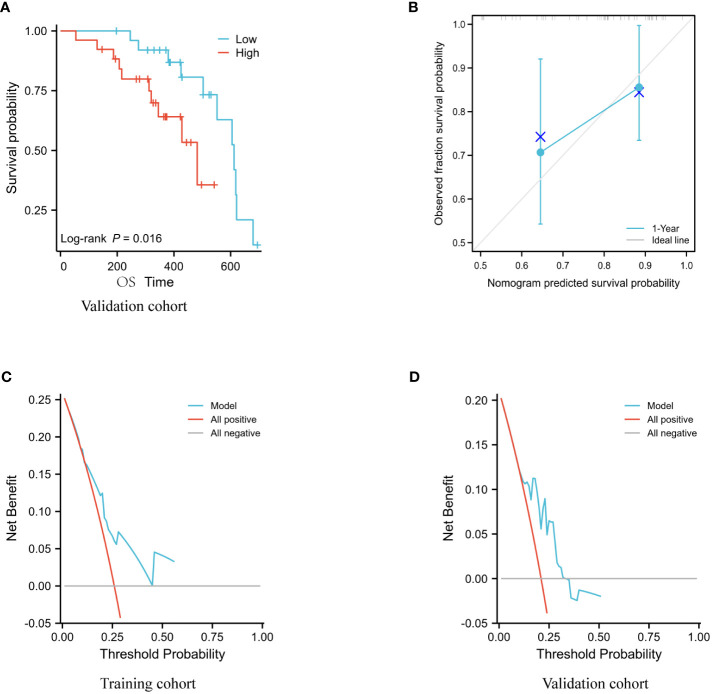
Model evaluation of the validation set. **(A)** Analysis using Kaplan-Meier of the association between OS and ALI in the validation cohort. **(B)** Validation of column line graph calibration plots for predicting 1-year OS in a group of hepatocellular carcinoma participants. **(C, D)** DCA analysis of the validation set and training set models.

Using an ALI of 36.5 from the training set as the cutoff, the validation set data revealed a significant difference in the overall survival rates of the two groups (**Figure 4A**). The prediction models for the training and validation sets were tested using calibration plots. The results of the calibration curves revealed that the predicted probabilities of the models were quite close to the observed probabilities, with only slight deviations (**Figure 4B**).

The C-index of the model for the training set was 0.720 (95% CI: 0.686-0.753, p<0.01) and for the validation set it was 0.722 (95% CI: 0.652-0.791, p = 0.0357).

The DCA results indicate that the Nomogram model developed in this study has strong predictive consistency and can more accurately predict the prognosis of immunotherapy-treated patients with advanced liver cancer in both the training and validation sets (**Figures 4C, D**).

## Discussion

Hepatocellular carcinoma (HCC) is one of the cancers with the highest morbidity and fatality rates worldwide, and the majority of patients are already in an advanced stage at the time of diagnosis (12). Previously, tyrosine kinase inhibitors such as sorafenib were the first-line treatment for advanced HCC, despite their limited efficacy. Immune checkpoint inhibitors, such as programmed cell death protein 1 (PD-1) and its ligand (PD-L1), have become one of the new standard of care for first- and second-line treatment of advanced hepatocellular carcinoma (12, 13) in recent years, as they continue to demonstrate good efficacy in the treatment of advanced hepatocellular carcinoma. Despite improvements in overall patient survival, a substantial minority of patients with advanced HCC continue to be inadequately managed. In addition, there is a dearth of established efficacy and prognostic indicators for immunotherapy patients with hepatocellular cancer.

In fact, immunotherapy appears to help just a subset of HCC patients; consequently, effective markers are required to discriminate and identify this subset. Given these principles, it is vital to have a deeper understanding of the function of prospective biomarkers. These variables, including programmed death ligand 1 (PD-L1) expression, tumour mutational load (TMB), microsatellite instability (MSI) status, gut microbiota, and a number of other markers, may be able to predict the outcome for a portion of patients, according to previous research (14, 15). However, they have drawbacks such as low specificity, costly testing, and inconvenient use.

Cancer and inflammation are intimately intertwined, and inflammation is not only associated with an increased incidence of cancer but also with a bad prognosis for individuals with tumours, according to previous research. Consequently, a number of inflammation-related indicators have been identified to have predictive value for cancer patients’ survival. NLR has been identified as a poor prognostic marker for a number of tumours (16, 17). Despite the fact that NLR reflects the systemic immune inflammatory response, cachexia due to chronic systemic inflammation may affect patient prognosis *via* BMI and serum albumin levels (18). Therefore, an ALI index that includes both of these factors may more accurately reflect the nutritional status and systemic inflammatory status of patients and may be able to predict survival outcomes in malignancies (19). ALI is more discriminating and predictive than inflammation-based markers alone (20). Jafri et al. (21) discovered that a low ALI was substantially and independently linked with a poor outcome in advanced NSCLC. In this study, a retrospective review of the clinicopathological features and survival outcomes of 98 patients with advanced hepatocellular carcinoma demonstrated that the ALI index in immunotherapy patients with advanced hepatocellular carcinoma had a more accurate prognostic ability. The validation sample of the 52 follow-up sample corroborated these findings in addition.

According to the examination of the ROC curve, ALI was more discriminating for OS than other clinical variables. This may be owing to its use of human BMI data, which more accurately represents the nutritional condition and systemic inflammation of the host (18, 22). In addition, we set the ALI threshold at 36.5, separated the patients into two groups, and did a Kaplan-Meier analysis, which revealed that the high ALI group was associated with a higher OS rate.

In our investigation, we determined the ALI threshold to be 36.5 based on the ROC curve, while earlier studies have utilised a somewhat wide variety of thresholds, including 18, 19.5 and 31.1 (12, 18, 21, 22). This may be a result of the various types and stages of tumours that have been researched. Depending on the type of cancer, the degree of inflammation can differ even at the same staging stage.

Based on the results of a multifactorial COX regression analysis, we screened independent prognostic factors for patients with liver cancer treated with immune checkpoint inhibitors and constructed column line plots of these factors, which were found to have better predictive index results (C-index: 0.720; 95% CI: 0.686-0.753; p<0.01). In addition, the calibration plots of the column line graphs demonstrated a close approximation to the ideal 45° line, indicating that the incidence rates predicted by the line graphs under this model were near to the observed incidence rates. Due to the restricted follow-up time of this study, the results of the column line plot forecast years are limited to one year at this moment. Conclusion: The Nomogram prediction model developed in this study can be used in clinical practise to assess the probability of advanced hepatocellular carcinoma in immunotherapy-treated patients, based on their clinicopathological characteristics, and is a valuable resource for patient prognosis decisions.

Inflammatory reactions have a significant role in the development of cancer, and numerous studies (18) have implicated inflammatory markers as prognostic markers in cancer patients. Multiple causes, such as tissue inflammation triggered by tumour growth or invasion, cancer itself, and the release of inflammatory mediators induced by leukocytes, contribute to systemic inflammation in cancer patients (23). Inflammatory marker readings have predictive relevance for cancer patients (24, 25) because systemic inflammatory responses are responsible for cancer growth, invasion, metastasis, and resistance to chemotherapy. Specifically, for patients treated with immune checkpoint inhibitors, neutrophil-produced cytokines and chemokines can promote angiogenesis and extracellular matrix remodelling (26), creating a favourable microenvironment for cancer growth and impacting the efficacy of immune checkpoint inhibitors (27). In addition, lymphocytes play a crucial role in antitumor immunity; they destroy malignant cells by recognising cancer cell antigens (28), and these markers may be relevant to the immunological status of patients and their immunotherapy efficacy (29). Previous research (30, 31) has demonstrated that the ALI score is a powerful prognostic and predictive marker for advanced NSCLC lung cancer patients treated with PD-L1 inhibitors alone but not in combination with chemotherapy. It appears to correlate more strongly with total patient survival than other widely used clinical indices (32). In this study, the clinical use of ALI as a prognostic marker in patients with advanced hepatocellular carcinoma is investigated for the first time. This is the first study to investigate the prognostic importance of ALI in patients with advanced hepatocellular liver cancer treated with PD1 inhibitors, to our knowledge.

It is the first study to investigate the predictive role of ALI in the use of immunotherapy in patients with advanced liver cancer, whereas some previous studies have discussed the influence of immunotherapy in patients with liver cancer based on a single factor; the predictive model built from clinical data of patients is closer to clinical practise; the AFP and ALI subgroup scores are practical indicators that are readily available in clinical practise; and the predictive model built from clinical data of patients is more closely aligned with clinical practise. This also ensures the Nomogram model’s use.

As immunotherapy for liver cancer has only been used extensively in the clinic in recent years, this study is a single-centre study and future validation based on populations in other centres is required; it is a retrospective study and cannot avoid selection bias; it is a single-centre study and future validation based on populations in other centres is required; it We require additional prospective research to corroborate our findings.

## Conclusions

ALI can be used as a prognostic indicator in individuals with advanced hepatocellular carcinoma who are receiving immunosuppressive medication. A low ALI is an independent risk factor for a shorter OS in hepatocellular carcinoma patients. Nomograms combining ALI and AFP can give clinicians with predictive information on liver cancer survival, hence providing some reference value for immunotherapy in advanced liver cancer patients.

## Data availability statement

The original contributions presented in the study are included in the article/supplementary material. Further inquiries can be directed to the corresponding author.

## Ethics statement

The studies involving human participants were reviewed and approved by the ethics committee of Henan Cancer Hospital. The patients/participants provided their written informed consent to participate in this study. 

## Author contributions

Contributions: I Article conception and design: QL, FM. (ii) Data collection and compilation: QL, FM. (iii) Data analysis and statistics: FM, JW. All authors contributed to the article and approved the submitted version.
