# Corrigendum: Advanced lung cancer inflammation index predicts survival outcomes of hepatocellular carcinoma patients receiving immunotherapy

**DOI:** 10.3389/fonc.2024.1431058

**Published:** 2024-06-10

**Authors:** Qian Li, Fei Ma, Ju feng Wang

**Affiliations:** ^1^ Department of Oncology, The Affiliated Cancer Hospital of Zhengzhou University & Henan Cancer Hospital, Zhengzhou, China; ^2^ Department of General Surgery, The Affiliated Cancer Hospital of Zhengzhou University & Henan Cancer Hospital, Zhengzhou, China

**Keywords:** advanced lung cancer inflammatory index (ALI), hepatocellular carcinoma, Immunotherapy, nomogram, prognosis

In the published article, there was an error in affiliation 1. Instead of “Department of Oncology, Zhengzhou University’s Affiliated Cancer Hospital, Henan Cancer Hospital, Zhengzhou, China”, it should be “Department of Oncology, The Affiliated Cancer Hospital of Zhengzhou University & Henan Cancer Hospital, Zhengzhou, China”.

Furthermore, there was an error in affiliation 2. Instead of “Department of General Surgery, Zhengzhou University’s Affiliated Cancer Hospital, Henan Cancer Hospital, Zhengzhou, China”, it should be “Department of General Surgery, The Affiliated Cancer Hospital of Zhengzhou University & Henan Cancer Hospital, Zhengzhou, China”.

The authors apologize for these errors and state that this does not change the scientific conclusions of the article in any way. The original article has been updated.

